# Prevalence of Hypertension in HIV/AIDS Patients on Highly Active Antiretroviral Therapy (HAART) Compared with HAART-Naïve Patients at the Limbe Regional Hospital, Cameroon

**DOI:** 10.1371/journal.pone.0148100

**Published:** 2016-02-10

**Authors:** Christian Akem Dimala, Julius Atashili, Josephine C. Mbuagbaw, Akam Wilfred, Gottlieb L. Monekosso

**Affiliations:** 1 London School of Hygiene and Tropical Medicine, London, United Kingdom; 2 Faculty of Health Sciences University of Buea, Buea, Cameroon; University of Sao Paulo Medical School, BRAZIL

## Abstract

**Background:**

Highly active antiretroviral therapy (HAART) has greatly reduced the morbidity and mortality of HIV/AIDS patients but has also been associated with increased metabolic complications and cardiovascular diseases. Data on the association between HAART and hypertension (HTN) in Africa are scarce.

**Objectives:**

Primarily to compare the prevalence of HTN in HIV/AIDS patients on HAART and HAART-naïve patients in Limbe, Cameroon; and secondarily to assess other socio-demographic and clinical factors associated with HTN in this population.

**Methods:**

A cross-sectional study was conducted at the Limbe Regional Hospital HIV treatment center between April and June 2013, involving 200 HIV/AIDS patients (100 on first-line HAART regimens for at least 12 months matched by age and sex to 100 HAART-naïve patients). HTN was defined as a systolic blood pressure (BP) ≥ 140 mmHg and/or diastolic BP ≥ 90 mmHg.

**Results:**

The prevalence of HTN in patients on HAART was twice (38%; 95% CI: 28.5–48.3) that of the HAART-naïve patients (19%; 95% CI, 11.8–28.1), p = 0.003. In multivariate analyses adjusted for age, gender, smoking, family history of HTN, and BMI-defined overweight, HAART was associated with HTN, the adjusted odds ratio of the HAART-treated versus HAART-naïve group was 2.20 (95% CI: 1.07–4.52), p = 0.032. HTN was associated with older age and male gender, in the HAART group and with BMI-defined overweight in the HAART-naïve group.

**Conclusion:**

The prevalence of hypertension in HIV/AIDS patients in Limbe stands out to be elevated, higher in patients on HAART compared to those not on treatment. Blood pressure and cardiovascular risk factors should be routinely monitored. Other factors such as diet, weight control and physical exercise should also be considered.

## Introduction

The introduction of highly active antiretroviral therapy (HAART) greatly reduced the morbidity and mortality due to HIV/AIDS, with patients experiencing longer and healthier lives [1,2]. These increases in life expectancy and quality have however coincided with the epidemiological transition characterized by an increase in non-communicable diseases including cardiovascular diseases (CVD). Despite the paucity of data on CVD in people living with HIV in Africa, a recent study estimated the prevalence of self-reported CVD risk factors in HIV in Africa at 12% [3]. Furthermore, as many as 18% of HIV-infected adults in Uganda were found to have sub-clinical atherosclerosis, which can be predictive of CVD disease [4]. A review of published literature by Bloomfield et al. (2014) suggests that in Low and Middle Income countries cardiovascular diseases such as heart failure, hypertension, coronary artery diseases and stroke amongst others are common and appear to be more frequent in the HIV-infected population [5]. A large study of 5,563 patients initiating HAART in Uganda by Mateen et al. (2013) found that as many as 27.9% were hypertensive, though the ten-year risk of CVD based on the Framingham risk score was relatively low, particularly in women [6]. HAART has increasingly been associated with metabolic complications such as dyslipidaemia, dysglyacemia and hypertension [7–13]. As such there is more global concern about an increased prevalence of HTN and cardiovascular diseases (CVD) in HIV/AIDS patients on HAART [14–19]. However, multiple differences have been observed between the USA and European countries and Cameroon (and other African settings) in the epidemiology of HIV, its demographics and the availability of antiretroviral drugs, amongst others. HIV prevalence rates are relatively higher (4.3% in Cameroon in 2011 [20]), the main method of transmission is heterosexual and more than half of people living with HIV/AIDS are women. And despite increasing overall access, antiretroviral therapy is still limited in the availability of drug groups such as protease inhibitors. The choice of treatment regimens is thus limited. We therefore conducted this study, given the specificities of HIV in Cameroon, and the limited data on the prevalence of HTN in HIV/AIDS patients and on the association between HAART and HTN. We had as objectives to: 1. compare the prevalence of HTN in patients on HAART and HAART-naïve patients at the Limbe Regional Hospital (LRH); 2. compare the mean blood pressure of these patients; 3. evaluate the factors associated with HTN in these patients; and 4. determine if there is an association between HAART and HTN while controlling for confounders. We hypothesized that HTN will be more prevalent in patients on HAART and that the association will persist even after controlling for confounders.

## Methods

### Study design, period and setting

This was a hospital-based cross-sectional study at the HIV treatment center of the Limbe Regional Hospital (LRH) which is a second-level referral hospital in the South West Region of Cameroon. Participants were enrolled over a period of 3 months.

### Participants and sampling

The study population was made up of HIV/AIDS patients receiving longitudinal care at the HIV treatment center of the LRH between April 2013 and June 2013. These participants were: 1. A group of HIV/AIDS patients who had never received HAART (HAART-naïve) randomly sampled from the HIV/AIDS patients attending the LRH during the study period; and 2. a group of HIV/AIDS patients on HAART selected by consecutive convenient sampling matched by age and gender to the HAART-naïve group.

As selection criteria, we included: patients aged 21 years and above who consented to take part in the study; and for the HAART group, patients who had taken HAART for at least 12 months. We excluded patients with a confirmed non-adherence to HAART for 6 months and above. Patients with known cardiovascular risk factors such as renal diseases and diabetes were excluded. All patients with pre-existing hypertension before HAART initiation, whether on anti-hypertensive medications or not were also excluded. Patients on oral contraceptives, corticosteroids or other medications that could affect blood pressure were excluded.

### Study procedures and Variables

All participants were subjected to a face-to-face interview and a physical examination. Data was collected using a standardized questionnaire. Information on socio-demographics, smoking habit, alcohol consumption, physical activity, family history of HTN, CD4 cell count (within past 6 months), duration of HIV infection and HAART (all considered as predictor variables) were obtained both from the interviews and the patients’ medical records.

The physical examination entailed measurement of the height, weight, body mass index (BMI), waist and hip circumferences, waist-to-hip ratio (WHR) and determination of the WHO clinical staging of HIV of each participant (all considered as predictor variables also). The blood pressure (BP) of each participant was measured and hypertension (the outcome variable) was diagnosed from the BP values.

### Data sources and measurements

Two BP measurements were taken on each participant on the right arm in a sitting position, with at least 5 minute intervals of rest between measurements. An electronic automated and clinically validated BP monitor (Omron M2, HEM—7121—E) with a suitable sized cuff (22–34 cm) was used. The mean BP value for each participant was calculated and the diagnosis of HTN was made according to the WHO criteria as systolic BP ≥ 140 mmHg and/or diastolic BP ≥ 90 mmHg [21]. As such the provisional diagnosis of hypertension was made for the first time during the examination since all participants with an earlier diagnosis of hypertension made by their primary health care physician or some other health care provider were excluded. Weight (to the nearest 0.5 kg) was measured using a weighing scale (BRN 9311). Participants were permitted to keep on light clothing. Height (in meters to the nearest 0.5cm) was measured using a stadiometer. Body mass index (BMI in kg/m2) was calculated as weight (kg)/[height (m) X height (m)]. BMI-defined overweight was considered as a BMI between 25 to 29.9 Kg/m^2^ and BMI-defined obesity as a BMI ≥ 30 Kg/m^2^. Waist circumference was measured midway between the iliac crest and the lower rib margin and the hip circumference was measured at the intertrochanteric level. Waist-to-hip ratio was calculated as waist (cm)/hip (cm) circumferences. WHR-defined abdominal obesity was considered as a WHR > 0.9 in men and WHR > 0.85 in women. Excessive alcohol consumption was based on intake of either more than three (two for women) standard glasses of wine per day or more than ten (five for women) local beers (1 local beer contains 28 g of alcohol) per week. Participants were classified as having ever smoked or not. Regular non-work related physical activity was considered in participants reporting at least 30 min of intense physical activity, once a week or more.

### Sample size considerations

The minimum sample size (N) was determined using the formula for comparing two proportions [22]. Using pre-study estimates of the prevalence of HTN in patients on HAART and HAART-naïve patients of 17% and 2% respectively [19], we thus needed 156 participants (78 HAART-experienced and 78 HAART-naïve patients). To account for potential non-response, 200 participants were selected (100 on HAART and 100 HAART-naïve).

### Data management and data analysis

The data collected was entered into and analyzed using Epi Info version 7 statistical software (CDC, Atlanta, USA). For objective 1, the prevalences of HTN in each category were compared using the Chi-square test. For objective 2, the mean of the BP values in each category were compared using the Student t-tests. For objective 3, age, gender, family history of HTN, alcohol consumption, smoking, overweight/obesity, CD4 cell count, duration of HIV infection were assessed for any significant association to HTN using the Chi-square test or the Fisher’s exact test (where the Chi-square test was not valid). A p-value < 0.05 was considered statistically significant. For objective 4, a logistic regression model was built to assess for the association between HAART and HTN while adjusting for all factors found to be associated to HTN, in bivariate analysis, with p-values < 0.25 [23].

### Ethical considerations

Ethical approval was granted by the Institutional Review Board of the Faculty of Health Sciences of the University of Buea (approval number: 2013/0083/UB/FHS/IRB) and administrative authorization by the Regional Delegate of Public Health South West region and the director of the LRH. Participants were exposed to minimal risk since all measurements done were non-invasive. Confidentiality, anonymity and privacy of all participants were guaranteed at all levels of this study. Written consent was provided by each and every participant.

Participants with a provisional diagnosis of HTN received on-site medical counselling (on lifestyle measures to manage elevated BP and the need for future observation) and were referred back to the attending physicians or specialists of the LRH for workup and long-term management. Participants with extremely elevated BP values were immediately referred to the emergency department of the LRH for appropriate care.

## Results

### Socio-demographic and clinical characteristics of the study population

Of the 200 participants, 100 were on HAART and 100 were HAART-naïve. The socio-demographic and clinical characteristics of the participants are summarized in Table 1. There was no significant difference between the HAART group and HAART-naïve group with respect to the mean age (40.2±8.0 years vs. 38.0±10.6 years), gender distribution (70% females in both groups), occupation and region of origin, except for the marital status.

**Table 1:** Socio-demographic and clinical characteristics of the participants.

Characteristic	All participants (n = 200)	HAART group (n = 100)	HAART-naïve group (n = 100)	P-value
***Age (mean ± SD*, *in years)***	39.1±9.4	40.2±8.0	38.0±10.6	0.106
***Females*, *n (%)***	140 (70%)	70 (70%)	70 (70%)	1.000
***Married*, *n (%)***	89 (44.5%)	52 (52%)	37 (37%)	**0.033**
***Unskilled occupation*, *n (%)***	26 (13%)	12 (12%)	14 (14%)	0.884
***North west region*, *n (%)***	88 (44%)	46 (46%)	42 (42%)	0.850
***BMI (mean ± SD*, *Kg/m***^***2***^***)***	24.1±2.9	24.8±2.8	23.5±2.8	**0.002**
***BMI-defined overweight (prevalence*, *%)***	40.5%	50%	31%	**0.006**
***WHR (mean ± SD)***	0.86±0.06	0.86±0.07	0.85±0.06	0.333
***Duration of HIV infection (mean ± SD*, *in months)***	34.7±39.2	66.5±30.9	2.9±8.9	**˂ 0.001**
***CD4 cell count (mean ± SD*, *cells/μL)***	308±237	501±225	197±160	**˂ 0.001**
***WHO clinical stage III*, *n (%)***	98 (49%)	52 (52%)	46 (46%)	**0.020**

**Fig 1:**
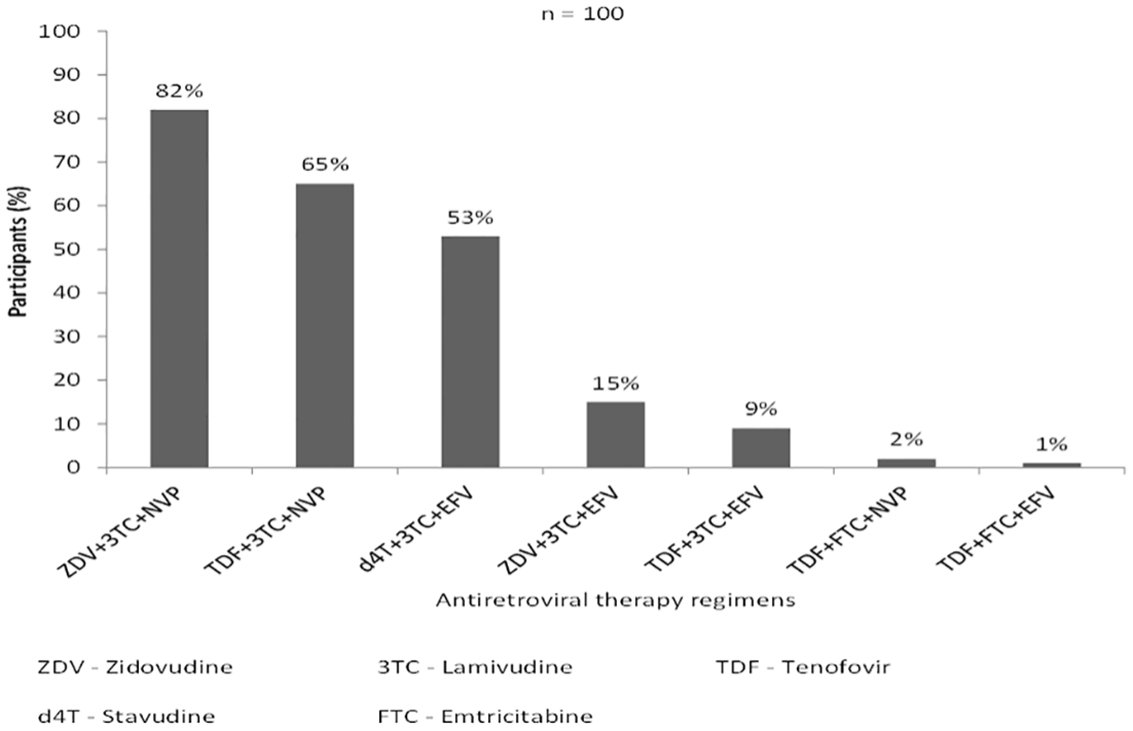
Antiretroviral regimens ever received by the participants.

There was a significant difference between the HAART and HAART-naïve group with respect to the mean BMI (24.8±2.8 Kg/m^2^ Vs. 23.5±2.8 Kg/m^2^), prevalence of BMI-defined overweight (50% Vs. 31%), duration of HIV infection (66.5±30.9 months Vs. 2.9±8.9 months), mean CD4 cell count (501±225 cells/μL Vs. 197±160 cells/μL) and the WHO clinical staging of HIV. There was no significant difference between these two groups with respect to the mean WHR (0.86±0.07 Vs. 0.85±0.06) and the prevalence of WHR-defined abdominal obesity (46% Vs. 43%). All the participants of the HAART group were on first-line antiretroviral therapy including drugs of the NRTI and NNRTI classes and some participants had received more than one first-line antiretroviral regimen. The mean duration of HAART was 58.6±28.5 months. The HAART regimens and the respective number of participants who ever used them since their initiation of treatment are shown in Fig 1.

### Prevalence of hypertension and mean blood pressure values

Table 2 summarizes the prevalence of hypertension and the mean SBP and DBP values in HIV/AIDS patients on HAART and HAART-naïve patients. The prevalence of HTN was significantly higher in the HAART group, 38% (95% CI: 28.5–48.3) than in the HAART-naïve group, 19% (95% CI, 11.8–28.1), p = 0.003. The mean SBP and DBP values of the HAART group (131±31 mmHg and 80±13 mmHg) were higher than that of the HAART-naïve group (125±19 mmHg and 77±12 mmHg), though not significantly (p = 0.060 and p = 0.118 respectively). No participant was on antihypertensive medications or any other medication known to either raise or lower blood pressure levels.

**Table 2:** Prevalence of hypertension and the mean blood pressure values.

Parameter	All participants (n = 200)	HAART group (n = 100)	HAART-naïve group (n = 100)	P-value
***Hypertension*, *n (%)***	57 (28.5%)	38 (38%)	19 (19%)	**0.003**
***(95% CI)***	22.4–35.3	28.5–48.3	11.8–28.1	
***SBP (mean ± SD)***	128±20	131±31	125±19	0.060
***DBP (mean ± SD)***	79±13	80±13	77±12	0.118

### Factors associated with hypertension

The various factors associated with HTN in these participants are summarized in Tables 3 and 4. In the HAART group, age above 40 and the male gender were the only factors significantly associated with having HTN. In the HAART-naïve group, only the BMI-defined overweight/obesity was significantly associated with having HTN.

**Table 3:** Factors associated with hypertension in the HAART group.

Factor	HAART group (n = 100)	Participants with HTN (n = 38)	P-value
***Age (in years)***			
*>40*	48	26 (54.2%)	**0.001**
*≤40*	52	12 (23.1%)	
***Gender***			
*Male*	30	17 (56.7%)	**0.012**
*Female*	70	21 (30.0%)	
***Family history of HTN***			
*Yes*	9	1 (11.1%)	**0.081**
*No*	91	37 (40.7%)	
***Smoking***			
*Yes*	6	4 (66.7%)	0.136
*No*	94	34 (36.2%)	
***Alcohol consumption***			
*Yes*	29	9 (31.0%)	0.359
*No*	71	29 (40.9%)	
***Physical exercise***			
*Yes*	26	9 (39.2%)	0.679
*No*	74	29 (34.6%)	
***BMI-defined overweight/obesity***			
*Yes*	50	22 (44%)	0.216
*No*	50	16 (32%)	
***WHR-defined overweight/obesity***			
*Yes*	46	17 (37.0%)	0.843
*No*	54	21 (38.9%)	
***CD4 cell counts***			
*<350 cells/μL*	8	3 (37.5%)	0.976
*≥ 350 cells/μL*	92	35 (38.0%)	
***Duration of HIV infection (months)***			
*> 30*	88	35 (39.8%)	0.323
*≤ 30*	12	3 (25.0%)	

**Table 4:** Factors associated with hypertension in the HAART-naive group.

Factor	HAART-naïve group (n = 100)	Participants with HTN (n = 19)	P-value
***Age (in years)***			
*>40*	30	9 (30.0%)	0.066
*≤40*	70	10 (14.3%)	
***Gender***			
*Male*	30	9 (30.0%)	0.066
*Female*	70	10 (14.3%)	
***Family history of HTN***			
*Yes*	2	0 (0%)	1.000
*No*	98	19 (19.4%)	
***Smoking***			
*Yes*	5	2 (40.0%)	0.219
*No*	95	17 (17.9%)	
***Alcohol consumption***			
*Yes*	23	5 (21.7%)	0.703
*No*	77	14 (18.2%)	
***Physical exercise***			
*Yes*	16	3 (18.7%)	0.978
*No*	84	16 (19.0%)	
***BMI-defined overweight/obesity***			
*Yes*	31	10 (32.3%)	**0.023**
*No*	69	9 (13.0%)	
***WHR-defined overweight/obesity***			
*Yes*	43	7 (16.3%)	0.547
*No*	57	12 (21.1%)	
***CD4 cell counts***			
*<350 cells/μL*	70	12 (17.1%)	0.470
*≥ 350 cells/μL*	30	7 (23.3%)	
***Duration of HIV infection (months)***			
*> 30*	4	0 (0.0%)	1.000
*≤ 30*	96	19 (19.8%)	

### Association between antiretroviral therapy and hypertension controlling for confounders

In multivariate analyses using logistic regressions we observed that being on HAART was significantly and positively associated with having HTN after adjusting for age, gender, family history of HTN, smoking and BMI-defined overweight and obesity. The adjusted odds ratio of having HTN comparing the HAART group to the HAART-naïve group was 2.20 (95% CI: 1.07–4.52, p = 0.032) (Table 5).

**Table 5:** Association between HAART and hypertension after controlling for confounders.

Factor and categories	Adjusted odds ratio*	95% confidence interval	P value
***Group*** *(HAART /HAART-naïve)*	2.20	1.07–4.52	**0.032**
***Age*** *(> 40 / ≤ 40 years)*	2.29	1.49–6.02	**0.002**
***Gender*** *(Male/Female)*	3.08	1.43–6.67	**0.004**
***Family History of HTN****(Yes/No)*	0.33	0.04–2.91	0.315
***Smoking*** *(Yes/No)*	2.10	0.53–8.29	0.291
***BMI-defined obesity*** *(Yes/No)*	2.48	1.20–5.10	**0.014**

*Odds ratio adjusting for all variables on the table.

### Antiretroviral Drugs

**Fig 2:**
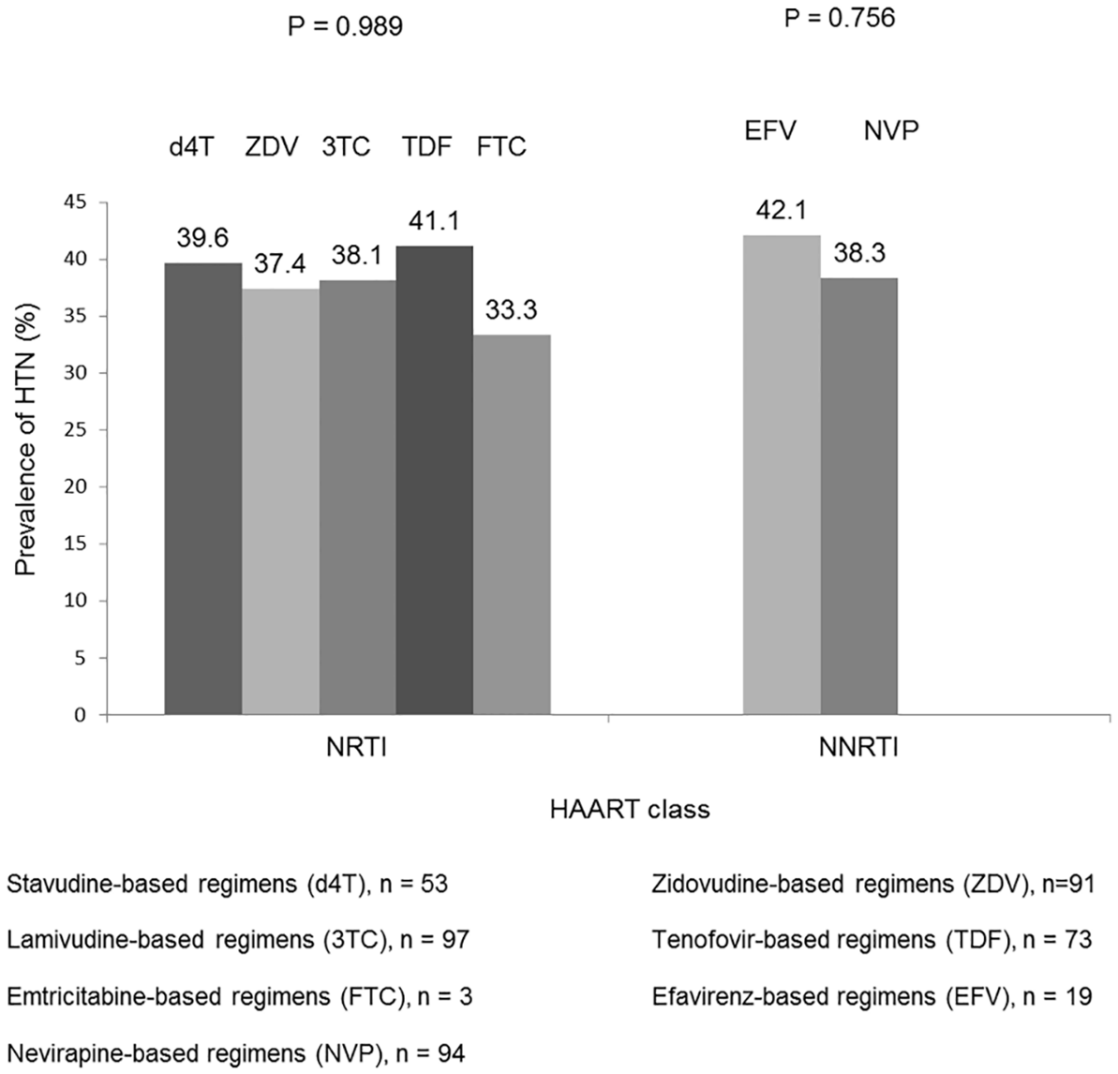
Prevalence of Hypertension in participants with respect to antiretroviral drug ever received.

The first-line HAART regimens ever used by the participants were noted (Fig 1). Most participants had used more than one HAART regimen. No particular NRTI or NNRTI was significantly associated with HTN in these patients (Fig 2).

## Discussion

In this study the prevalence of HTN was significantly higher in patients on HAART compared to HAART-naïve patients. HAART was significantly and positively associated with having HTN even after adjusting for confounders. These findings concurred with that of several studies which showed a higher prevalence of HTN with HAART [13,17–19,24], thus supporting the fact that HAART could possibly be linked to HTN in these patients. However this is contrary to the findings of other studies which showed no significant difference in the prevalence of HTN between patients on HAART and HAART-naïve patients [25,26] or even a lower prevalence of HTN with NNRTI’s use [27]. This discrepancy in findings could be attributed to factors such as the difference in geographical locations, study settings, clinical characteristics of the study population and even study designs.

The observed prevalence of HTN in our participants on HAART (38%) was higher than that found by other studies [13,16,26]. This is possibly because our participants had a higher mean duration on HAART (58.6±28.5 months) compared to the participants of the above studies. This is supported by the findings of the Multicenter AIDS Cohort Study which suggested a link between the duration of HAART and BP, demonstrating that prolonged HAART (defined as 2–5 years in duration) was independently associated with development of HTN, whereas HAART of less than 2 years in duration was not [17]. However, contrary to our study which as predominantly composed of females, all the participants of the Multicentre AIDS Cohort Study were males. It is worth noting that that our observed prevalence of hypertension in these HIV/AIDS patients could be lower than it actually is, given the fact that we excluded patients who were already diagnosed with hypertension before being placed on HAART. Also no participant received a definitive diagnosis of hypertension based on the measurements of blood pressure from our study. Those with elevated blood pressure values were referred to the attending physician of the health care facility for further investigation.

The mean SBP and DBP values of our participants on HAART were higher than that of HAART-naïve participants though not significantly. This is similar but not in total accordance with the findings of previous studies [13,26] in which the SBP and DBP values of the participants on HAART were significantly higher than that of the HAART-naïve participants. Nevertheless, these mean SBP and DBP values of our participants on HAART were much higher than that of the participants on HAART of the former studies possibly still because of the greater duration of HAART in our participants. However, the mean SBP and DBP values in our study were lower than those of participants in a retrospective study conducted in Italy [9], despite a higher mean duration of HAART in our participants. This is possibly because our participants were just on first-line therapy including NRTI’s and NNRTI’s with no PI while participants of the former study were on antiretroviral therapy including a PI (Indinavir). It is well documented that PI are associated with HTN [28].

As for the factors associated with HTN, age above 40 and the male gender were significantly associated with HTN in the HAART group while BMI-defined overweight/obesity was significantly associated with HTN in the HAART-naïve group. This is in line with the results of a recent study [13] in Cameroon conducted in a setting similar to that of our study. On the other hand, factors such as family history of HTN, smoking, alcohol consumption and physical inactivity were not significantly associated with HTN in both groups. This is possibly because our data was based on self-reports. It is widely believed that in such reports, respondents might be tempted to present themselves in a more favorable way in what is termed “social desirability”. This is with respect to the assessed lifestyle patterns of alcohol consumption, smoking and physical exercise. Also reports on family history of HTN were based on verbal confirmation from the participants and not from well documented medical records. Low CD4 count and duration of HIV infection were as well not associated with HTN, similar to the finding of other studies [15,16,18] with prospective cohort, retrospective cohort and case-control study designs respectively. This possibly means that irrespective of the study design, these factors are not involved in the causal pathway between HAART and HTN. The BMI-defined overweight/obesity and the WHR-defined abdominal obesity were not significantly associated to HTN in the HAART group. This is contrary to the results of other cross-sectional studies [19,26] which showed a positive correlation between waist-hip ratio and HTN. As such prospective cohort studies will be best suited to monitor the effect of HAART-induced anthropometrical changes such as elevated WHR, WHR-defined abdominal obesity and BMI-defined obesity on BP. In any case our study supports the fact that HAART could be significantly associated with HTN in Africans and it is possibly linked to the HAART-induced metabolic alterations such as hyperlipidaemia as earlier demonstrated in a study conducted in Cameroon [12].

Nevertheless, our study is limited by its cross-sectional design, restricting any inference about causality. Cohort studies are best indicated for monitoring BP changes induced by HAART among these patients. Further studies will need to be prospective, excluding participants with hypertension prior to HAART initiation. Also our study was not designed to determine which particular antiretroviral drugs or regimens could be associated with HTN since many participants used more than one antiretroviral regimen. We also did not include HIV-negative controls as in other studies [18,28]. This study was conducted in a regional hospital in Cameroon and generalizability of results to other hospitals in other regions of the country may therefore not be possible. Also, it was not possible to distinguish primary from secondary hypertension. Finally, the study did not control for unmeasured potential confounders such as diabetes, renal disease and dyslipidemia.

In conclusion, we observed that HIV/AIDS patients on HAART were twice more likely to have HTN and higher BP values than patients not on HAART in Limbe even after adjusting for potential confounders. Age above 40 and the male gender were associated with HTN in patients on HAART and BMI-defined obesity in the HAART-naïve patients. Routine monitoring of HTN and other cardiovascular risk factors should be encouraged in HIV/AIDS patients in Limbe. Also, traditional hypertension control measures such as diet, weight control and physical exercise should be considered in this population.
